# Expressions of Multiple Neuronal Dynamics during Sensorimotor Learning in the Motor Cortex of Behaving Monkeys

**DOI:** 10.1371/journal.pone.0021626

**Published:** 2011-07-06

**Authors:** Yael Mandelblat-Cerf, Itai Novick, Eilon Vaadia

**Affiliations:** Department of Medical Neurobiology, Institute for Medical Research Israel-Canada, Faculty of Medicine, The Edmond and Lily Safra Center for Brain Sciences, The Hebrew University, Jerusalem, Israel; Université Pierre et Marie Curie, France

## Abstract

Previous studies support the notion that sensorimotor learning involves multiple processes. We investigated the neuronal basis of these processes by recording single-unit activity in motor cortex of non-human primates (Macaca fascicularis), during adaptation to force-field perturbations. Perturbed trials (reaching to one direction) were practiced along with unperturbed trials (to other directions). The number of perturbed trials relative to the unperturbed ones was either low or high, in two separate practice schedules. Unsurprisingly, practice under high-rate resulted in faster learning with more pronounced generalization, as compared to the low-rate practice. However, generalization and retention of behavioral and neuronal effects following practice in high-rate were less stable; namely, the faster learning was forgotten faster. We examined two subgroups of cells and showed that, during learning, the changes in firing-rate in one subgroup depended on the number of practiced trials, but not on time. In contrast, changes in the second subgroup depended on time and practice; the changes in firing-rate, following the same number of perturbed trials, were larger under high-rate than low-rate learning. After learning, the neuronal changes gradually decayed. In the first subgroup, the decay pace did not depend on the practice rate, whereas in the second subgroup, the decay pace was greater following high-rate practice. This group shows neuronal representation that mirrors the behavioral performance, evolving faster but also decaying faster at learning under high-rate, as compared to low-rate. The results suggest that the stability of a new learned skill and its neuronal representation are affected by the acquisition schedule.

## Introduction

In sensorimotor learning, the brain remaps a sensory instruction to a motor command when interactions with the environment require it. For example, in a commonly used adaptation paradigm, a force-field is used to perturb arm reaching movements by pushing the hand away from the target, causing the hand to deviate from its planned trajectory. Many studies have shown that humans and monkeys adapt easily to force-field perturbations, and that their trajectories straighten with practice [*1*]–[*5*]. To re-optimize the movement [*6*] and minimize kinematic error and effort [*7*], the brain needs to correctly anticipate the force-field and modify the motor command accordingly [*8]*, [9**].

Motor learning has been shown to progress at different adaptation rates as a function of the experimental paradigm. However, the learning curves during both fast short-term learning [*10]*, [11**] and slow long-term learning [*12]*, [13**] are mostly characterized by a fast stage of improvement in performance, followed by a slower and more subtle stage of improvement [*14*]. These two stages are hypothesized to reflect dynamical neuronal subsystems with multiple timescales [*15]*, [16**].

Studies have pointed to the potential advantages of learning at multiple processes, suggesting it may allow flexibility [*17*] and act as a mechanism for the functional hierarchy of neuronal systems [*18*]. Smith et al. 2006 [19] explained several phenomena observed during short-term force-field adaptation by a multi-processes model in which fast processes respond quickly to new environments but have poor capabilities for retention, while slower processes respond more slowly to new environments but have stronger retention capabilities.

There is an extensive body of literature on the patterns of motor learning generalization, including the effect of adaptation to perturbed movements in one direction on subsequent movements that differed with regard to direction [4], [*20*]–[*24*], speed and amplitude [*25]*, [26**], workspace location [*27]*, [28**], and even movements of the other arm [*29*]. In particular, adaptation to force-field perturbation showed only limited and narrow generalization to other directions, mostly affecting movements in nearby directions [*21]*, [30**] . Interestingly, this range of generalization may be affected by environmental complexity [*31*] and time [*32*].

In this study we tested the hypothesis that the acquisition, retention and generalization of sensorimotor skills are mediated by multiple neuronal processes, which differ in their dynamics, following Smith et al. 2006 [19]. We manipulated the number of perturbed arm movements to a selected direction, which were interleaved with unperturbed movements to other directions, inducing a fast or slow learning. We found support to our hypothesis by the different dynamics of learning and its neuronal representations that evolved by the different practice schedules.

## Results

**Figure 1:**
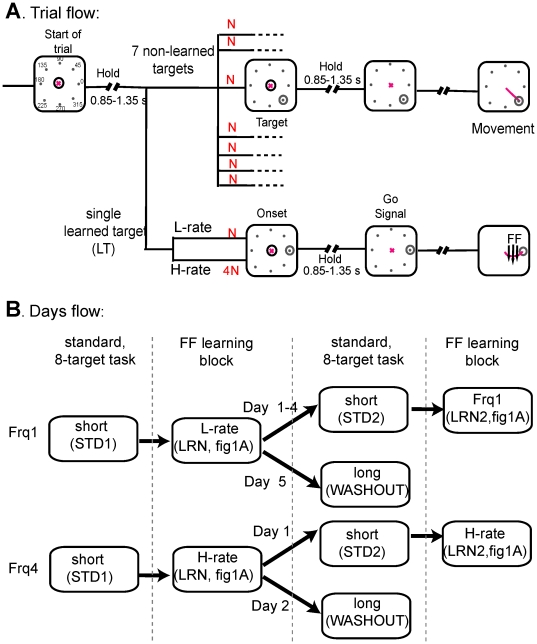
Experimental design. (A) example of a trial flow (left to right) during a learning epoch under “L-rate” and “H-rate” practice schedules. During the first delay period the monkey held the robotic arm in the center without moving it. The monkey kept holding at the central circle after target onset for an additional delay and moved after the go signal. In the figure the learned target is at 0° and the force field (FF) is clockwise (parallel arrows). If the lit target was the selected learned target (lower row), then the force-field was applied when movement was initiated, otherwise (upper row) movement was executed under standard conditions. Under the L-rate schedule the learned target appeared as often as the other targets (N), and under the H-rate schedule, four times more frequently (4*N). (B) **Recording day flow:** all days started with standard trials (center-out reaching movements to eight directions) followed by a learning epoch. From the first day until the day before last (fourth day under the L-rate, first day under the H-rate) the learning epoch was followed by a second standard epoch and then ended with a second learning epoch. The last day of the learning set (fifth day under the L-rate, second day under the H-rate) consisted of only three epochs in which learning was followed by a long standard epoch (“washout”).

Local adaptation to force-field, in two different practice schedules, induced differences in dynamics of behavioral and neuronal changes. In the first practice schedule, the rate in which the learned target appeared was relatively low ( “L-rate”), whereas in the second, the learned target appeared four times more ( “H-rate”), as depicted in figure 1. To reach a plateau of best performance, the same learned target and the same force-field direction were learned for several consecutive days (“learning set”). The high rate practice resulted in a faster acquisition.

Data includes 7 learning sets for the L-rate (5 from monkey R and 2 from monkey O) and 4 learning sets for the H-rate (3 from monkey R and 1 from monkey O).

### Behavioral findings

**Figure 2:**
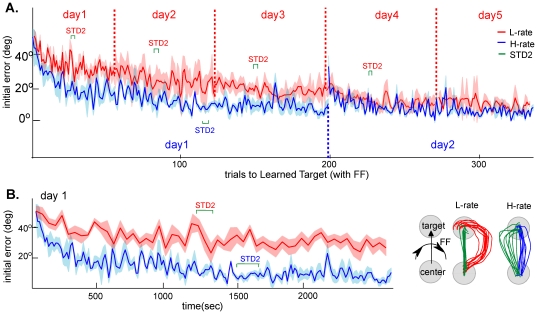
Movement kinematics show slow or fast improvement during L-rate and H-rate learning sets, respectively. (A) Initial directional deviation of movements to the learned target (LT), as a function of the number of trials to the learned target along the learning days of L-rate (red) and H-rate (blue). (B) Initial directional deviation as a function of time during the first day of the L-rate (red) and H-rate (blue) learning sets. Right: examples of trajectories to the learned target on the last 10 force-field trials (red, blue) and during the STD2 (green) on the first learning day. Note that performance under the H-rate showed complete adaptation on the first day with minimal deviation under the perturbation and large aftereffects in the standard trials that followed adaptation. In contrast, under the L-rate condition it took 4-5 days to reach similar performance. Green brackets denote the range in which the STD2 epoch interfered with learning at different learning sets (standard trials are not shown). Shaded areas denote SEM.

During adaptation, monkeys gradually learned to compensate for a perturbing force-field and achieved straighter movements. To quantify learning we compared directional deviations of trajectories at the initial stage of the movement (150 ms after movement onset, before sensorimotor feedback may result in correction of movement) during trials to the learned target under force-field. Deviations were normalized to the force-field direction such that positive errors were in the force-field direction, and negative errors counter to the force-field. Figure 2A depicts the initial directional deviations as a function of trial number under both H-rate and L-rate along their learning sets: five days under L-rate (red, reported previously in Mandelblat-Cerf et al 2011 [33]) and two days under H-rate (blue). Note that (i) trials presented here are a concatenation of learning trials from LRN and LRN2 epochs, in which the monkeys initiated a movement (either successful or not) and were not aborted prior to the go-signal; (ii) green brackets denote approximately the point where the concatenation occurred. Namely, the point where STD2 epoch interfered with the learning in the different sets, and (iii) the number of trials for each day (1-5 for L-rate and 1-2 for H-rate) was truncated to the number that was actually performed on that day across sets.

Previous studies in our lab [*3*] showed that when the learned target was the only target used during learning, adaptation to the force-field took only tens of trials. In this study, learning was significantly slower. Under the H-rate condition, learning reached a plateau within 100 force-field trials (Figure 2A, blue, day1). Under the L-rate condition, learning was much slower and a plateau performance, with an average angular deviation of approximately 10°, was reached only after five days and over 200 trials (Figure 2A, red, day5).

At the end of day one, after a similar learning duration (∼2500sec, Figure 2B), performance under the H-rate was significantly better than under the L-rate (t-test, p<0.01). This was expected since there were more trials to the learned target under the H-rate than under the L-rate during this time interval. When comparing performance as a function of the number of force-field trials (figure 2A), the difference between errors in the last trials of the L-rate (47–56) and the same trials of H-rate was smaller, but errors under the L-rate were nevertheless significantly larger (t-test, p<0.05). Therefore, the H-rate schedule facilitated learning as compared to the L-rate, even for the same number of trials. Figure 2B (right panel) also illustrates movements toward the learned target (from bottom to top) with a clockwise force-field (left to right). The plots depict the last 10 force-field trials of the first day under the L-rate (red) and the H-rate (blue) and the trajectories during STD2 (no force-field, green). Note that under H-rate practice schedule, but not under L-rate, movements in STD2 were curved counter to the force-field direction (“aftereffects”), reflecting the advanced stage of learning under H-rate as compared to L-rate.

Next, we examined different periods that interfered with the learning process in which force-field trials were not executed and compared the effect on performance under either the L-rate or the H-rate. In order to properly compare the two conditions we primarily focused on the late stages of learning, when performance had reached similar and relatively stable levels. Specifically, we compared day four and day five of the L-rate learning set to the later trials of day one and day two of the H-rate.

**Figure 3:**
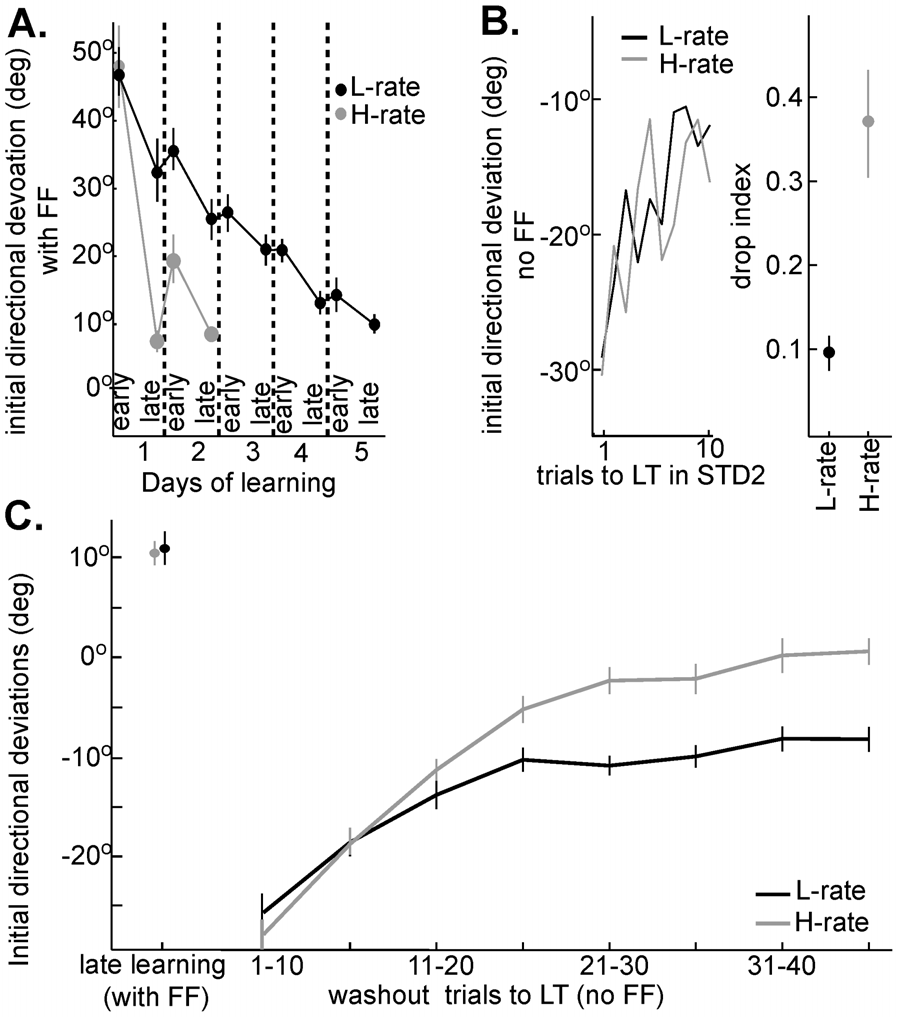
Learning under high rate practice schedule (H-rate) is more labile to interference than learning under the low rate (L-rate). Performance in all plots is measured by the initial directional deviations of the hand. (A) **Effect of the overnight pause**: Performance on the first and the last 5 force-field trials in each learning day (L-rate: days 1-5 in black, H-rate: days 1–2 in grey). In the L-rate performance gradually improves over the days with almost no overnight drops between consecutive learning days, whereas under the H-rate it shows a significant overnight drop (t-test, p<0.01) between the first (10°) and second day (20°). (B) **Effect of the standard epoch** (STD2): The plots show the aftereffects of learning on standard trials in STD2 (left) and the effect of these trials on the subsequent continuation of learning (right plot). The left plot shows that the aftereffects gradually decreased in both the L-rate (black) and the H-rate (grey). However, the right plot shows that the drop in performance between force-field trials before and after STD2 (right) was significantly larger under the H-rate than the L-rate (t-test, p<0.01). (C) **Effect of washout**: Performance without force-field (aftereffects) after learning under L-rate practice schedule (black) was washed out less than performance after learning under H-rate (grey), which was washed out completely. The figure shows that performance on the last 10 force-field trials of the L-rate and H-rate learning sets were similar (∼10°, ANOVA p>0.5) and resulted in similar aftereffects when switching to standard trials (∼−25°, ANOVA p>0.5). The aftereffects gradually decreased (less negative values in the plot), but from trial 15 until the end of washout the aftereffects in the L-rate were larger than in the H-rate. The aftereffects in the last 10 trials of the washout were significantly different from zero in the L-rate (t-test, p<0.01) but not in the H-rate (t-test, p>0.5).

The longest pause between the two learning epochs was the overnight time between two consecutive learning days, followed by the STD1 epoch in the subsequent days. Figure 3 shows that although learning was faster under the H-rate schedule, the improvement was less stable. Figure 3A depicts the average initial deviations over the first and last 5 force-field trials in each day along the learning set, for the L-rate (black) and the H-rate (grey). The figure shows that under the H-rate, performance dropped overnight, and the first few movements under force-field of day two were again curved (the deviations late in day one of the H-rate were small (∼10°), and the trajectories in early trials of the following day were significantly more curved (∼20°)). Then, as learning continued in day two, the movements gradually improved to return to the plateau level of day one, after about 500 s or 20 trials (see figure 2A). In contrast, under the L-rate, performance continued to slowly and gradually improve in the following days (Figure 3A) with almost no overnight drops in performance, reaching a plateau on day five. Note that deviations in the last 5 trials of day four in the L-rate were almost as small as those at the end of day one under the H-rate, but performance the next day (day5, last day) did not drop.

We then compared the strength of adaptations in the L-rate and the H-rate by looking at overnight aftereffects. To do so, we examined the aftereffects in STD1 trials, selecting the learning days that reached a plateau of 10–15° initial error. Accordingly, we compared aftereffects during STD1 on day five of the L-rate and day two of the H-rate. The aftereffects (deviations counter to force-field direction, data not shown) did not change significantly along the STD1 epoch in either case (comparing aftereffects in the first and last 3 trials of STD1, t-test, p>0.1), but were higher in the L-rate as compared to the H-rate (two-sample t-test, p<0.05) with average aftereffects of −21° under L-rate and −15° under H-rate. The reduced aftereffects after the overnight pause under the H-rate suggest that the drop in performance of the force-field trials resulted from the overnight decay of the learned task and not washout during STD1.

The second pause in learning we examined was introduced by the short epoch of standard trials (STD2), again by comparing epochs of similar performance in the H-rate vs. the L-rate. We compared day one of the H-rate and day four of the L-rate, which was the first L-rate day in which performance in the last 5 force-field trials prior to STD2 was similar to the performance of these trials under H-rate (ANOVA, p>0.5). Figure 3B (left plot) shows that aftereffects during the STD2 epoch were apparent and gradually decreased (less negative) in both the L-rate (black) and the H-rate (grey). Surprisingly, the influence of this decrease on learning differed between the practice schedules. We computed the “Drop Index” by comparing performance immediately following the return to learning (first 3 trials of LRN2 epoch) to the last 5 force-field trials prior to STD2 (see Methods). Note that in order to capture the relearning effect we reduced the number of sampled trials after STD2 to 3. Apparently, as depicted in Figure 3B (right plot), the drop under the H-rate was significantly larger than under the L-rate (t-test, p<0.01). Therefore, the slower learning under L-rate practice schedule was less susceptible to this short washout.

Last, the third period we examined was the long standard epoch (washout), which was employed at the end of the L-rate and the H-rate learning sets (Figure 3C). Under both practice schedules, performance under force-field prior to washout was similar (t-test, p>0.5), with about 10° deviations in the initial movement direction. When the force-field was removed, the aftereffects in movement to the previously learned target were also similar (∼−25°, t-test, p>0.5) and gradually decreased as washout progressed. However, from trial 15 there was a growing divergence in the progression of the washout between the H-rate (grey) and the L-rate (black). Aftereffects following the H-rate practice were eventually completely washed out and reached zero deviations (t-test, p>0.5). In contrast, the aftereffects following the L-rate remained significantly larger than H-rate (t-test, p<0.01) with ∼−9° deviations in the last 10 trials of washout. Note that during washout, in both cases, the learned target was presented at the same rate as other targets (like the L-rate schedule), regardless of whether learning was under the L-rate or H-rate practice schedules. These experimental data support the notion that a practice schedule which induces a slower process, also generates more robust savings than a faster process, which renders it less vulnerable to interference. However, in both cases, aftereffects were diminished the following day (aftereffects were statistically not different from zero, t-test, p>0.1, not shown), suggesting the washout of L-rate learning continued offline during the night.

### Neuronal Data

The sample totaled 851 isolated single-cell activity records (645 from monkey R, 206 from monkey O) from an indeterminate number of actual different neurons during 43 daily training sessions (7×5 = 35 L-rate sessions and 4×2 = 8 H-rate sessions). Criteria for selecting these cells are described in the Methods section (2346 other recorded cells did not pass these criteria and were not used in analyses). Note that we did not attempt to record the same cells on different days, or determine how the composition of the sample of isolatable neurons may have changed from day to day. Therefore, comparisons over days were done by daily averaging the changes in cell activity over the population data.

For each cell, we characterized the relationship among the preferred movement direction (PD), the direction of the learned target, and the force-field direction. To do this we calculated each cell's “nPD,” which defines the angular distance of the cell's PD from the learned target, signed by the force-field direction. A positive value denotes nPD in the direction of the force-field, and negative in the opposite direction (counter to force-field). Cells with nPD in the range of 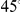 to 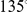 were defined as "co-FF" and cells with nPD in the range of 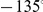 to 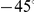 were defined as "counter-FF."

To study the neuronal basis of the behavioral differences between the high and low practice schedules that we reported above, we examined changes in firing rates of these subgroups of cells along the learning sets. More specifically, we computed the firing rate of each cell in a 500 ms interval around movement onset, from 200 ms before to 300 ms after. We then examined the firing rate in standard trials to the learned target (STD1) as compared to the firing rate during learning under force-field perturbations.

In line with previous reports from our lab [*3]*, [33**], no significant consistent changes were found in the subgroups of cells with PDs near the learned target or PDs in the opposite direction (not shown) whereas counter-FF cells increased their activity during force-field learning and co-FF decreased.

**Figure 4:**
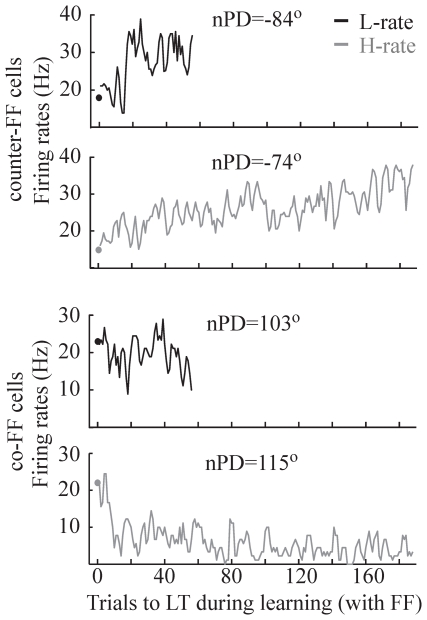
The activity of four different single cells in the L-rate condition (black) and H-rate condition (grey) on the first learning day as a function of trial number. The figure depicts (from top to bottom): a counter-FF cell recorded under the L-rate (nPD = −84°), a counter-FF cells recorded under the H-rate (nPD = −74°), a co-FF cell recorded under the L-rate (nPD = 103°) and a co-FF cell recorded under the H-rate (nPD = 115°). For each cell (trace) the figure shows average firing rate across standard trials to the learned target (circle) and firing rates along the learning trials to the learned target.

Figure 4 demonstrates activity of four different single cells that were recorded either under L-rate (black) and H-rate (grey), with nPDs either in the counter-FF range (upper traces) or the co-FF range (lower traces). Each trace depicts the average firing rate in the standard epoch of the first learning day (day1, STD1, circle) and firing rates along the subsequent learning trials of this day. Note that learning in day1, both under H-rate and L-rate, started from a naive state. However, under H-rate, behavioral performance improved in day1 until it reached a plateau, while under L-rate it did not. The figure shows increased activity in both counter-FF cells, whereas only the co-FF cells under H-rate, but not under L-rate, showed decreased activity.

**Figure 5:**
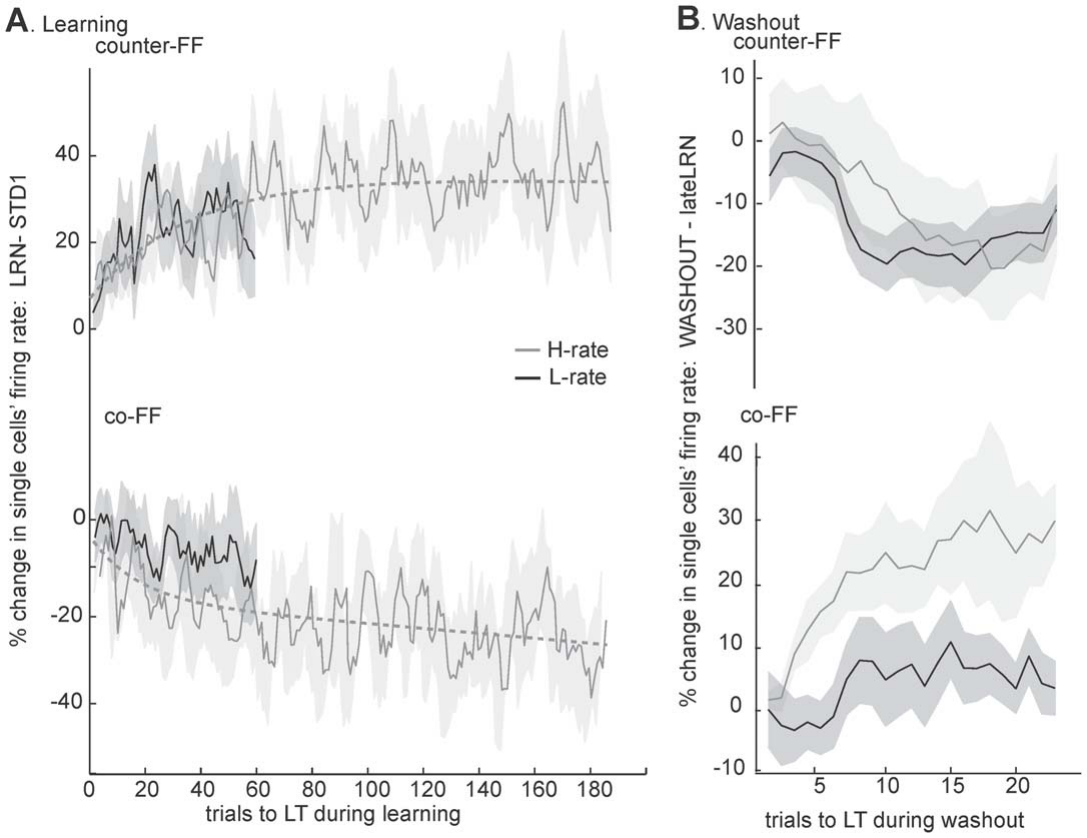
Different dynamics in counter-FF and co-FF cells under L-rate and H-rate as a function of trial number. Figure depicts analyses of cells with PDs in the range −135°<nPD<−45° (counter-FF, top) and range 45°<nPD<135° (co-FF, bottom) (A)Dynamics of changes in activity during adaptation trials to the learned target (LT) relative to standard trials in the first learning day. Counter-FF cells showed increased activity (p<0.01) under L-rate (number of cells = 29) and H-rate (number of cells = 17), with similar magnitudes (p>0.5) in the two conditions, for the same number of trials. In contrast, co-FF cells showed a significant decrease in activity only under H-rate (number of cells = 21, p<0.01), whereas changes under L-rate (number of cells = 36) were not significant (p>0.1). (B)Changes in activity in movements to the learned target along washout (standard trials) relative to activity on the last 10 force-field trials. Counter-FF cells decreased their activity significantly (p<0.01) and similarly under H-rate as compared to L-rate (number of cells = 18 and 33, respectively. p>0.5). Co-FF cells showed a significant increase only under H-rate (number of cells = 13, p<0.01) but not under L-rate (number of cells = 23, p>0.1). Shaded areas denote SEM.

We then assessed the changes in activity during learning, relative to the standard epoch, across all counter-FF and co-FF cells recorded in day1 of L-rate and H-rate learning sets. Figure 5A shows the activity during the same time interval (2500 s) under H-rate (grey, over 180 trials) and L-rate (black, 56 trials). The counter-FF cells (upper plot) increased their activity substantially relative to standard, both under the L-rate and the H-rate practice schedule. The changes, as a function of number of trials, reached the same degree of increased activity in trials 47–56 (the last trials under L-rate, ANOVA p>0.5) and followed the same two-exponential curve (grey dashed line, see Methods). Namely, after experiencing the same number of trials, whether under the L-rate or the H-rate, neural activity exhibited similar changes. However, under H-rate, much more trials were executed, as compared to L-rate, for the same time interval. In those subsequent trials the activity of counter-FF cells slightly further increased, reaching a significantly higher level of activity in the last 20 trials (160–180) as compared to trials 47–56 (ANOVA, p<0.05). Thus, for the same time interval, the firing rates of counter-FF cells increased faster and to a higher level under the H-rate than the L-rate (as a function of time and not trial number).

When examining modulations of co-FF cells (lower plot) we found a significant decrease in activity only during the H-rate. The fitted curve for the H-rate (dashed grey line) showed a transient fast decrease followed by a slower one, whereas the changes in activity under the L-rate were non-significant and slowly decreased with values above this curve. The slow dynamics of changes in co-FF cells under L-rate is in line with our previous reports, showing that these changes accumulated to a significant decrease in activity only in 4^th^ day of learning (Figure 4 in Mandelblat-Cerf et al, 2011 [33]). This difference in timescales between H-rate and L-rate was obviously even more pronounced when observing changes as a function of time rather than number of trials (not shown).

Altogether, changes in counter-FF cells were trial dependent, and changes in co-FF cells were both trial and time dependent. Most of the increases in counter-FF cells along learning occurred approximately in the first 50 trials, independent of time, while the decreases in activity of co-FF cells seemed to depend on the practice schedule and to follow the rate at which trials appeared.

Next, we examined the extent to which the changes in activity were reversed during washout when the aftereffects gradually decreased (as shown in figure 3C). Figure 5B shows the activity along washout (trials without force-field) to the learned target relative to the average firing rate late in learning, in the last 10 force-field trials. Note that during the washout epoch, the learned target appeared as often as other targets, regardless of the previous practice schedule. Clearly, the counter-FF cells (top traces), which increased during learning, decreased similarly during washout after both the L-rate and the H-rate practice schedules and reached a 20% decrease late in the washout (t-test p<0.01). Note that the similar dynamics of counter-FF cells during washout, after H-rate practice as compared to L-rate practice, are consistent with their similar increase in activity as a function of number of trials during learning. In co-FF cells, the reverse process was expected to show increased firing rate during washout. This is indeed what we observed (Figure 2B, lower traces). However, here the reversals were different between the L-rate and the H-rate conditions. The washout effect was significant only after learning under H-rate practice schedule (t-test, p<0.01), whereas after L-rate, the increase was much smaller (ANOVA p<0.01) and did not reach significance. The results may therefore suggest that the timescale of increase in the activity of co-FF cells during washout followed its timescale during learning, which was dependent on the practice schedule (in our experiment, H-rate or L-rate). More specifically, its resistance to washout was negatively correlated with the frequency of trials during practice and the resultant pace of learning.

**Figure 6:**
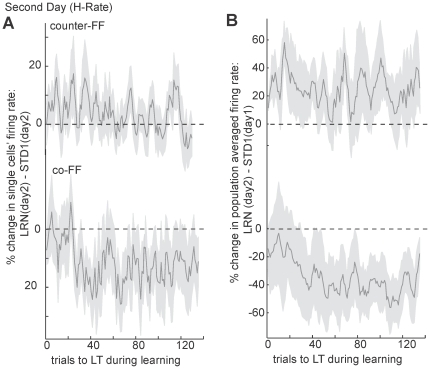
Neuronal activity during the second day of H-rate practice schedule. Figure depicts analyses of cells with PDs in the range −135°<nPD<−45° (counter-FF, top) and range 45°<nPD<135° (co-FF, bottom). (A) Percent of change in single cells firing rate along force-field trials from standard trials to the learned target (LT) show non-significant changes in activity of counter-FF cells (upper trace) but a significant decrease of activity in co-FF cells (bottom trace). Number of cells  = 31; shaded areas denote SEM. (B) Percent of change in the averaged firing rates across counter-FF and co-FF cells as compared to the averaged firing rate of cells with PDs in the same range during pre-learning standard trials of day1. The plot shows that counter-FF cells maintain their increased activity, while activity co-FF cells is initially similar to standard and only later decreases. Data includes 4 learning sets. Shaded areas denote SEM.

We then examined learning effects across days of the learning set. As shown in Figure 3A, learning under L-rate practice schedule gradually improved along five days with very little overnight drops in performance. However, under the H-rate, where the behavioral improvement was much faster and reached a plateau at the end of the first day, a significant overnight drop in performance was observed on the second day, which then improved to converge again to the same plateau level. We examined the neuronal correlates of the behavioral changes under the H-rate on these two days, and found that counter-FF cells did not show any additional increase in activity (Figure 6A, top trace) on the second day whereas co-FF showed a decrease (Figure 6A, lower trace). It is therefore possible that the overnight drop was mainly caused by a gradual fading of changes in co-FF cells, which were re-acquired on the second day. This notion was strengthened by extraction of the cumulative effect of learning. We compared the averaged population activity during learning in the second day to the averaged population activity that was recorded prior to learning (i.e. STD1 of day1). As depicted in figure 6B, the activity of counter-FF cells shows a relatively steady activity (as expected from figure 6A) of an increased activity in a similar magnitude that was observed in the first day (compare to figure 5A), suggesting that the activity of Counter-FF cells did not drop overnight. However, the activity of co-FF cells was initially similar to pre-learning STD1 (20 trials not significantly different from zero, t-test, p>0.3) and only later significantly decreased (last 20 trials, t-test, p<0.01).

The substantial overnight drop occurred only under the H-rate, but not the L-rate, suggesting that overnight reversed changes in co-FF cells occurred when the practice schedule provoked fast learning and therefore induced fast and pronounced changes during the learning epoch.

Last, we inspected how the second short standard epoch (STD2), which appeared between the learning epochs, influenced cell activity. We compared the activity of counter-FF and co-FF cells before (last 5 trials) and after (first 3 trials) STD2. The changes were statistically non-significant, but their trend was in line with the behavioral results (Figure 3B, right): counter-FF cells showed similar decreases under the L-rate and the H-rate whereas co-FF cells only increased under the H-rate (not shown). This trend supports the notion that the larger drop in performance after STD2 under the H-rate relates to the different dynamics of co-FF cells.

Altogether, the results indicate that changes in the activity of counter-FF cells occurred mostly early in learning and were “practice-dependent” since they increased (during learning) and decreased (during washout) as a function of number of trials, independent of time. Learning-related changes in co-FF cells and their stability appear to depend on the practice schedule; the faster the activity changed during learning, the more labile it was.

### Generalization – behavioral and neuronal data

To test the effect of the practice schedule on generalization we studied the trajectories to the other seven non-learned targets, which were executed (without force-field) throughout the learning epochs. Of particular interest were movements to target at −45°, where generalization was the strongest. These analyses are described below.

In order to compare the L-rate and the H-rate, we examined, as in previous analyses, advanced stages of learning after reaching plateau levels where initial directional deviations of the hand under force-field were similar (ANOVA, p>0.5; i.e., day four and day five for the L-rate, and late trials of days one and two for the H-rate).

**Figure 7:**
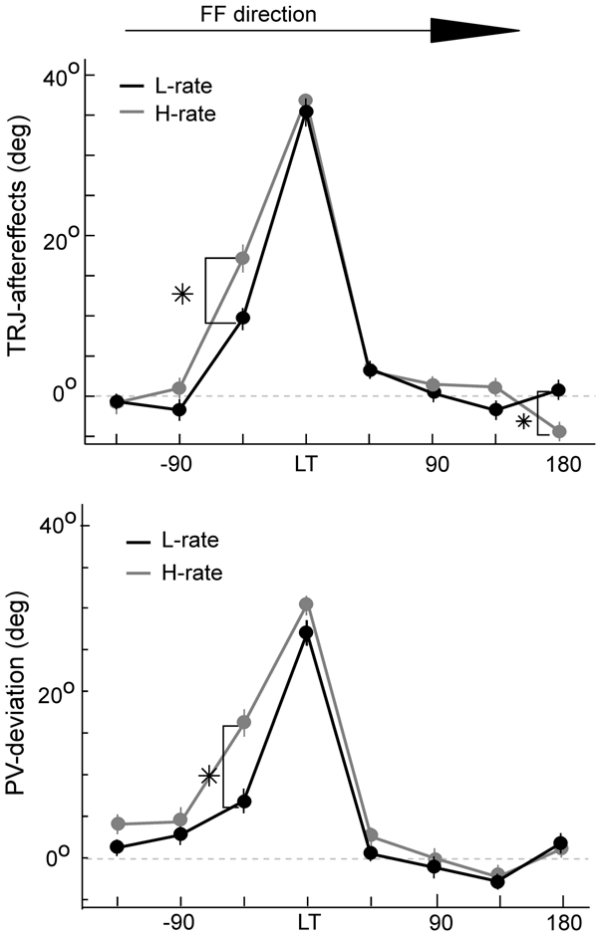
Generalization of local force-field learning is asymmetrical around the learned target (LT) and dependent on the practice schedule. The effect is measured by the initial directional deviations of the trajectories (TRJ-aftereffects, upper plot) and the corresponding population vectors (PV-deviations, lower plot). The figure shows the TRJ-aftereffects (upper) and PV-deviations (lower) from targets around LT during L-rate (black) and H-rate (grey) learning, taken at late stages of adaptation. Deviations from target at -45° were almost 10° larger under H-rate than L-rate (p<0.01). Note that for illustration purposes, positive values were assigned to deviations counter to the force field direction. In addition, measuring TRJ-aftereffects to the LT was only possible in STD2 after the first epoch of learning trials. Number of cells  = 298 for late days of L-rate, number of cells  = 156 for H-rate. Error bars denote SEM. Asterisks denote 1% significance.

The first analysis (Figure 7) compared movements in unperturbed trials (that immediately followed force-field trials) to each target under L-rate and H-rate, as a function of the angular distance of that target from the learned target (L-rate analysis was previously reported in Mandelblat-Cerf et al., 2011 [33]). The figure depicts the averaged initial directional deviations of trajectories (“TRJ-aftereffects,” upper plot) for each target under the two practice schedules and the corresponding population vectors deviations (lower plot, PV-deviations, using optimal linear estimator of Salinas and Abbott 1994 [*34*]). Note that for each target, population vectors were generated for each movement to this target by the neural sample in that daily recording session. Then, data was pooled across recording sessions.

Since we only analyzed trials from the advanced stages of learning where performance under the L-rate and the H-rate was similar (Figure 3A), as expected, there were no significant differences between H-rate and L-rate aftereffects on the trajectories (36° and 37°, respectively) and the population vectors (27° and 30°, respectively) to the learned target. However, there was a significant effect of the practice schedule on generalization. This effect was mostly expressed in movements to the target at −45° (the target adjacent to the learned target in a direction counter to force-field) where generalization was significantly larger under H-rate than under L-rate practice, with aftereffects on the trajectories of 17° vs. 9°, respectively (t-test, p<0.01, denoted by asterisk). Similarly, the corresponding PVs deviated from the target at −45°, with significantly larger directional errors of about 16° under the H-rate vs. 7° under the L-rate (t-test, p<0.01, denoted by asterisk). Significant, but considerably smaller, aftereffects on trajectories emerged both in the L-rate and the H-rate in movements to +45° target (∼5°, t-test, p<0.01) and only in the H-rate to the target at +180° (∼−4°). Movements to other targets did not show any systematic aftereffects on trajectories or PV-deviations, and on average did not differ from zero.

**Figure 8:**
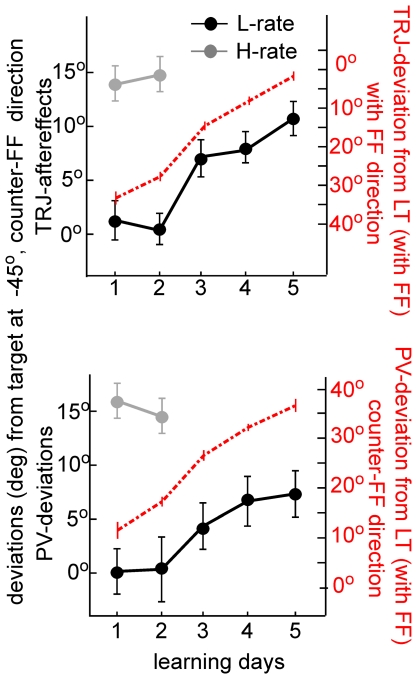
The generalization effect in movements to the target at −45° is correlated with adaptation. The developments of TRJ-aftereffects (upper plot) and PV-deviations (lower plot) from the target at −45° along learning days. Deviations were significant in the later days of the L-rate (black) learning set and in both days of H-rate (grey). Note that the gradual development along L-rate was closely related to the decreased deviations in movement to the learned target (dashed red line, upper plot) and to the increased deviations of the corresponding PVs (dashed red line, lower plot). number of cells  =  851. Error bars denote SEM.

Since generalization and the differences between the L-rate and the H-rate were primarily expressed in movements to the target at −45°, we performed further analyses to study the dynamics of the development and decay of generalization to this direction under the two practice conditions. To do so, we studied the trajectory aftereffects and population vector deviations throughout learning, and not only in its late stages. Figure 8 displays the aftereffects (upper plot) and population vectors (lower plot) as a function of the learning day. Note that as in Figure 7, we only included trials to target at −45° that immediately followed force-field trials. Aftereffects under the L-rate (black line) gradually changed to deviate from the target at −45° in a counter-FF direction, and became significant on days 3–5 (t-test, p<0.01). This generalization effect was highly correlated in time with improvement in performance of the learned movements to the learned target (dashed red line, red y-axis on the right). Under the H-rate, aftereffects reached a maximal and much higher deviation on the first day (grey line). Analyses of the population vectors to the target at −45° and the learned target (Figure 8, lower plot) showed similar dynamics, with a gradual deviation counter to the force-field direction along the L-rate (reaching significance on days 4–5, t-test, p<0.01) and much higher deviations on the first day of the H-rate. Finally, population vectors in movements to the learned target also reflected similar temporal dynamics, showing a gradual increased deviation counter to the force-field direction.

**Figure 9:**
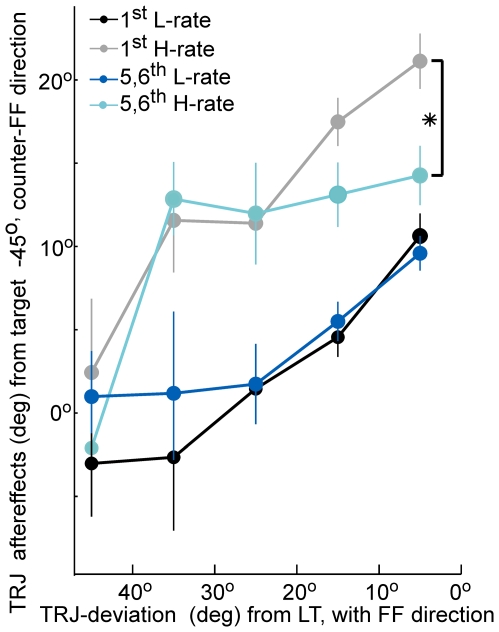
The generalization effect under H-rate is larger than under L-rate but more labile. The figure depicts the trajectory aftereffects (no force-field) to the target at −45° as a function of the trajectory deviations to the learned target under the force-field (in bins of 10°) for trials that immediately followed a force-field trial (1^st^-trials, L-rate in black, H-rate in grey) and aftereffects after 5–6 trials (5,6^th^-trials, L-rate in blue, H-rate in cyan). Note that for any directional deviation from the learned target smaller than 40°, the immediate aftereffects under H-rate (grey) were significantly larger than under L-rate (black). However, note also that under H-rate the large aftereffects significantly decay within 5 trials (asterisk), while under L-rate they do not show such decay. Error bars denote SEM. Asterisks denote 1% significance.

To study the temporal stability of generalization we directly examined aftereffects on a trial-by-trial basis in all incidences in which a non-perturbed movement to the target at −45° immediately followed a learning trial to the learned target (“1^st^-trials”) as compared to incidences where these unperturbed movements appeared only later, at trials 5 or 6 after a learning trial (“5,6^th^-trials”). Note that in the case of 5,6^th^-trials there were only unperturbed trials between the learning trial and the examined unperturbed trial to target at −45°. Therefore, we chose to look at trials 5 and 6 rather than later trials, since under H-rate schedule the chances of not having a learning trial for more than 6 subsequent trials are extremely small. Figure 9 shows the relation between trajectory deviations to the learned target under the force-field (x-axis, in bins of 10 degrees) and average aftereffects to the target at −45° (y-axis) for the 1^st^-trials (L-rate in black, H-rate in grey) and 5,6^th^ trials (L-rate in blue, H-rate in cyan). Data included trials from all learning epochs throughout the learning set. Note that along the x-axis the early stages of learning were mostly represented by large trajectory deviations from the learned target, while deviations late in learning were mostly smaller (as expected from the learning curves, Figure 2). A statistically significant effect of learning trials on the following movements (1^st^-trials) was observed: smaller deviations from the learned target were correlated with larger aftereffects in the following movements to the target at −45° (p<0.01). Furthermore, these aftereffects were significantly higher under the H-rate than the L-rate (t-test, p<0.01 for each of the bins with TRJ-deviation<40°). However, the large aftereffects under the H-rate, that were observed immediately following a trajectory deviation to the learned target smaller than 20°, decreased significantly within 5 trials after the perturbation (denoted by asterisk). In contrast, under the L-rate such a decrease was not evident and the aftereffects were similar regardless of their lag from a learning trial (t-test, p>0.5).

In sum, the generalization analyses indicate that at all stages of adaptation the immediate generalization to the target at −45° under the H-rate was larger than under the L-rate and consequently resulted in a larger asymmetry of the generalization counter to the force-field direction. Interestingly, the higher level of generalization, which evolved during the high rate practice (H-rate), decayed faster than the L-rate.

## Discussion

We present a study of local adaptation to force-field (FF) perturbation in which the perturbed movements to the selected target (“learned target”) were interleaved with unperturbed movements to other target locations at a high or low rate (H-rate and L-rate, respectively). Our findings show that (1) adaptation was faster and exhibited more pronounced generalization under the H-rate practice schedule than the L-rate; (2) learning under H-rate was more labile and subject to interferences; (3) generalization was asymmetric around the learned target, in counter force-field direction under the two practice scedules, but asymmetry was larger under the H-rate; (4) generalization was more labile under the H-rate; (5) two subgroups of cells which were previously described by our group [3], [33] exhibited different relationships to the adaptation progress. Specifically, the counter-FF cells, with preferred directions that “pushed against” the perturbation, changed as a function of number of trials, independent of time (“practice-dependent process”) and mostly early in learning. In contrast, the co-FF cells, with preferred directions that assist the perturbation direction, changed at a timescale that depended on the practice schedule - their learning pace was significantly slower under L-rate as compared to H-rate, and depended on the number of trials and the time it took to perform them (“schedule-dependent process”). The implications of these findings are discussed below.

The comparison of the two practice schedules showed that practicing under H-rate schedule, in which the time between consecutive force-field trials was short, facilitated adaptation. This result seems to contradict previous studies that demonstrated facilitation under long inter-trial-intervals in different learning tasks [*35*]–[*38*]. In particular, force-field adaptation showed trial-to-trial facilitation under longer inter-trial-interval [*39]*, [40**]. However, in these studies, the inter-trial-intervals were simply pauses in time, whereas in our study the time intervals between learning trials were interleaved with unperturbed movements to other directions that may have undermined the benefits of these long intervals for learning.

The faster learning and higher lability in the H-rate schedule may be a combined result of some or all the following reasons:

First, the interleaved unperturbed trials may induce anterograde and retrograde interference effects on movements to the learned target [*41]*, [42], [21**]. Thus, the larger number of interleaved unperturbed trials under L-rate could induce higher interference as compared to H-rate, which could slow down the learning pace and modulate the lability.

Second, evidence suggests that the brain can switch between different motor commands as a response of context switching between different environmental conditions [*43*]–[*45*]. It was previously shown that mixed practice schedules of several tasks leads to a slower acquisition but a more stable representation of the learned tasks [*46*]–[*48*], an effect that is known as the "context interference effect." Accordingly, our learning paradigm could elicit a strategy of context switching between the perturbed trials to the learned target and unperturbed trials to other targets, resulting in slower but more stable learning under L-rate, which is in line with our results. It is also possible that the frequent appearance of the learned target in the H-rate could induce an increased ability to use the learned target as a context cue to anticipate the perturbation [*49*]. However, our results show that as the local learning progressed, the aftereffects to a non-learned target increased (Figure 8). Since context switching does not predict such an effect, it is likely that the brain does not rely solely on context switching to learn our task.

Third, since the probability of appearance of perturbed reaches to the learned target during H-rate was four times larger than that of any of the other targets, it is possible that at higher rate statistics were learned faster. The effects described above of anterograde, retrograde and context interferences may contribute to this process. Under H-rate, the interferences are weaker, facilitating acquisition of the statistics, and increasing the validity of the learned adapted model. Thus, in the H-rate condition just a few consecutive perturbed trials are sufficient to increase the probability that the adapted model indeed holds, and only a few unperturbed trials – that the original model should resume. This possibility is consistent with the notion of optimal control, predicting that during local adaptation, the brain attempts to globally minimize some cost function [*50]*, [51**].

Fourth, a major difference between L-rate and H-rate learning sets is that the task design allows several periods (four nights) of post-practice consolidation in the L-rate, but only one in the H-rate paradigm. The impact of these periods on the dynamics of learning is controversial. Some studies have shown that the fragility of a motor memory trace is reduced in time [*52]*-[54**], with or without sleep [*55*], while others have failed to reproduce these results [56]–[*58*]. This contradiction may be the result of an inability to retrieve the learned skill rather than an inability to form a stable memory trace. Therefore, it is possible that the higher lability of the learning under H-rate schedule is a result of the fewer post-practice periods.

### Neuronal Changes under L-rate and H-rate practice schedules

The pace of changes in firing rates of co-FF cells (termed “schedule dependent”) was correlated with the learning pace, while the pace of counter-FF cells (termed “practice-dependant”) was not. We suggest that the difference in the dynamics of these two groups of cells allows the brain to maintain a good performance, while achieving flexibility in consolidation that is advantageous for motor behavior. Our results suggest that the increased activity of the counter-FF cells is directly related to performance (evaluated by the trajectories' kinematic properties), since most of the improvement in performance occurred early in learning, just like most of the changes in these cells. In contrast, it appears that the decrease in activity of co-FF cells may lag behind the improvement in performance, as shown in the L-rate learning. Therefore, the co-FF cells are probably related to other aspects of movement optimization and maybe also to consolidation. It is most likely that these cells represent the part of the neuronal population that is more sensitive to the time and/or events between learning epochs. This may originate from sensitivity to the interferences imposed by the interleaved unperturbed trials and/or sensitivity to post-practice periods (nights) during learning. This sensitivity then results in manipulation of the strength of consolidation as a function of the practice schedule.

Consequently, we suggest that it is the schedule-dependent process, and not the practice-dependent process, that accounts for the weaker retention after H-rate practice. Our experimental data further support this notion by showing a complete washout after H-rate but not L-rate (Figure 3C) as well as different decay profiles of the co-FF cells. In contrast, the decay profile of the counter-FF cells was similar, regardless of the previous practice schedule (Figure 5B). In addition, we observed a large overnight drop of the learned behavior under the H-rate but not the L-rate (Figure 2A, 3A). As discussed in the results section, the behavioral improvement that followed this overnight drop may be related to a re-adaptation of the schedule-dependent cells.

Our results put strength to the proposal that neuronal processes with multiple timescales underlie sensorimotor learning [*19*] in which one process evolves slowly but retains information well, while the second evolves faster but shows weaker retention. The faster changes of the schedule-dependent cells during H-rate learning decay faster, and may be the cause of the less stable retention of the learned motor skill. When the practice schedule induced slower learning, these cells show slower changes during learning with better retention. This could also be a result of the difference afforded by the overnight consolidation periods in H-rate (one night) versus L-rate (4 nights) [53], [54], [*59*].

**Figure 10:**
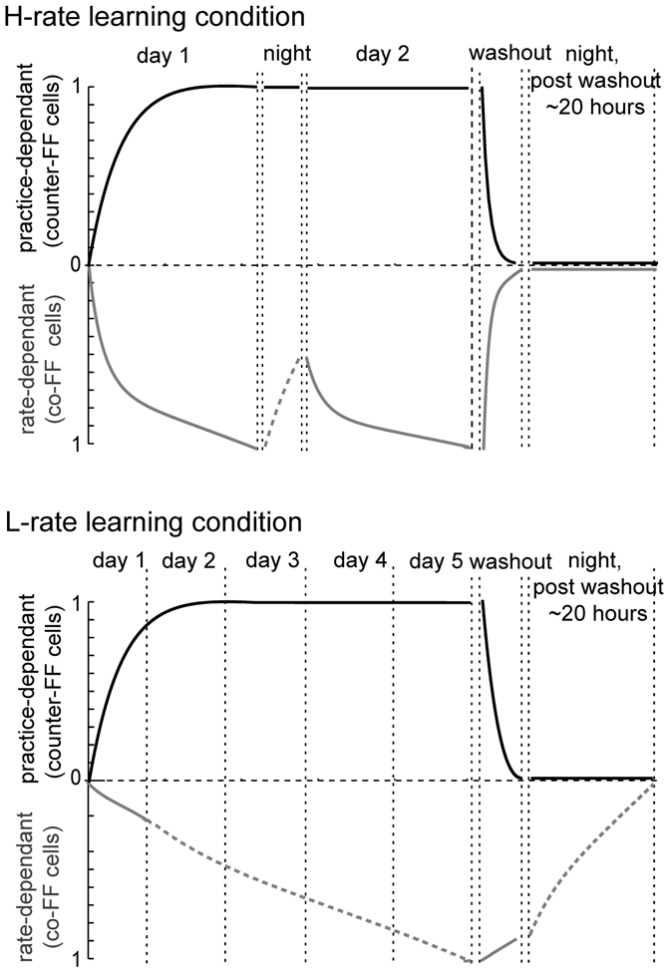
A qualitative scheme of the schedule-dependent and practice-dependant neuronal processes can account for the observed behavioral changes during learning. The evolvement of the practice-dependent process that reflects the observed dynamics of counter-FF cells (black) and the schedule-dependent process reflecting the co-FF cells (grey). The scale is set arbitrarily from zero to one to represent a transfer from a naive state to a fully adapted state. The behavior reaches plateau only when the two processes reach one. Solid lines are based on observed results and dashed lines - speculations. During H-rate (upper plots) both processes reach the value of one on the first day. An “overnight drop” in the value of the schedule-dependent process re-emerged on the second day, corresponding to the observed overnight drop in performance (as in Figure 3A) and the change of only the co-FF cells in the second day (as in Figure 6). The washout results in a reversed change (as in Figure 5B) of both processes to zero, reflecting the diminishing aftereffects (as in Figure 3C). During the L-rate (bottom plots), the practice-dependent process evolved as in the H-rate, but it took more time, since trials appeared at a lower rate. The development of the schedule-dependent process was slower. The curve is only speculative to illustrate the slow development. The washout in the L-rate reversed only the practice-dependent process (as in Figure 5B, black). We assume that the schedule-dependent process was reversed overnight since the observed results did not show aftereffects on the following day.

A unified scheme to explain how neuronal adaptation processes serve as the basis of the observed behavioral adaptation is shown in Figure 10. The figure depicts the neuronal changes during H-rate (top) and L-rate (bottom) learning sets of the practice-dependent cells (black) and the schedule-dependent cells (grey). The changes are modeled between zero to one, where zero is the naive state and one is the maximal change, such that learning is complete when both groups of cells reach the value of 1. To obtain the curves that reflect each group's dynamics from naive to plateau performance, we normalized the fitted curves for the changes of co-FF and counter-FF cells during the first day of H-rate learning (Figure 5A). Details of the relation of the scheme to the data are further elaborated upon in the figure's legend.

### Generalization

The design of this study differs from previous studies of adaptation to force-field perturbation. Local adaptation (learning trials to a single target) with interleaved unperturbed movements (to all other targets) allowed us to dynamically evaluate the level of generalization by examining unperturbed movements during learning.

Previous studies have demonstrated limited generalization of adaptation [*21]*, [60], [61**]. In agreement with these studies we have previously reported [*34*] that generalization is indeed limited. However, we have also shown an increased generalization effect in the direction that counteracts the force-field; namely, in the direction that the hand must push to compensate for the force-field and reach the learned target. Interestingly, we found here that the behavioral and neuronal aftereffects in this same direction were substantially stronger under the high rate practice.

This result supports the notion of optimal control, suggesting that the acquisition and retention is facilitated when the likelihood of the adapted model is higher and thus it becomes more cost effective to assume it is the valid one (as discussed above). Hence, generalization in the direction of the (preferred) adapted model is facilitated in the H-rate as compared to L-rate schedules.

At the same time, we found that in the H-rate schedule, generalization decayed faster (Figure 9), in line with the higher lability of the learned skill. This result suggests that generalization can be rapidly modulated within few trials.

In conclusion, this study introduced two practice schedules that demonstrated how the brain learns at two different paces. These different paces were represented by differences in the dynamics of changes in the activity of subpopulations of cells. The high temporal correlation of the behavioral phenomena and changes in these subpopulations may be suggestive of a neural substrate for the different paces of learning, the lability of the adapted models, and the strength of generalization.

## Materials and Methods

### A. Animals, recordings and behavioral task

Ethics Statement: Animal care and surgical procedures complied with the US National Institute of Health (NIH) Guide for the Care and Use of Laboratory Animals. The study was approved by the Institutional Committee for Animal Care and Use at the Hebrew University, permit number MD-78-03-3.

Details of animal welfare and steps taken to ameliorate suffering were in accordance with the recommendations of the Weatherall report, "The use of non-human primates in research".

Animals were kept in common yards with enrichment devices. For reinforcement learning reasoning, they were kept under food restrictions during the week. Drops of juice (usually Gerber enriched with baby formula) were provided as a reward for task success. Monkeys enjoyed weekends of full feeding and at all times were not deprived of water. A veterinarian inspected them weakly and performed routine tests. All procedures were sterile and under anesthesia, with pain relievers.

Two monkeys (Macaca fascicularis, ∼4 kg) were chronically implanted with a microelectrode array (Cyberkinetics Neurotechnology Systems, Foxborough, MA) on the contralateral arm region of the motor cortex, under anesthesia and aseptic conditions.

#### Behavioral task

Two monkeys used a robotic arm (Phantom Premium 1.5 High Force, SensAble Devices, Cambridge, MA) to control the movements of a cursor on a video screen in a two dimensional plane. Prior to surgery, monkeys were trained to perform a default eight-target center-out reaching task (“standard” trials). The phantom manipulated a cursor on the screen to move from the starting point at the center of the screen (origin) to a visual target in a delayed go-signal paradigm. The trial sequence and recording day flow are shown in Figure 1. Figure 1A depicts the trial flow from left to right during learning. Each trial began when the monkey moved the cursor to the origin (central circle). After a variable hold epoch of 0.85–1.35 s, a target appeared at one of the eight possible positions, which were uniformly distributed in a circle 4 cm from the origin (Figure 1A, second column). After an additional 0.85–1.35 s hold epoch the origin disappeared (go signal, third column), prompting the monkey to move to the target in less than 0.8 s (fourth column). This generous time constraint allowed relatively natural reaching movements. After another 0.4 s when the monkey kept the cursor still in the target, a liquid reward was delivered.

The perturbation is defined by a combination of a single target (the "learned target”) and force-field direction (clockwise or counter clockwise). The robot-generated force-field pushed the hand perpendicular to its current velocity in a counterclockwise or clockwise direction. Given the components of the observed trajectory in the horizontal plane (*x* and *y*), the velocity-dependent force-field was generated using the following equation:
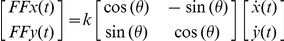



where FF*x* and FF*y* are the robot-generated forces at time sample *t*, *k* = 8 Ns/mm, θ = ±90°, and 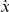
and 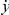
are the components of the hand velocities in the horizontal plane. Thus, the perturbation is velocity dependant and the hand was pushed only while it was moving. Furthermore, the monkeys experienced the perturbation only while reaching to the learned target and not when returning the hand to the origin. While movements under standard conditions were typically straight to the target, when the perturbation was introduced, trajectories to the learned target were initially curved (as depicted in figure 2 and in Mandelblat-Cerf et al 2011). To reduce the curvature the monkeys needed to compensate for the force-field. This could be carried out by learning to push against the force-field properly while moving to the target. Note that since the force-field was velocity dependant it was not fixed. Therefore, the pattern of the applied compensatory force should have been adapted accordingly.

A *learning set* consisted of several learning days during which the same perturbation (Learned target and force-field direction) was applied. On each day, during standard epochs, the sequence of targets was randomly chosen from a uniform distribution and was executed without any perturbation. During learning epochs, targets continued to appear randomly but the rate at which the learned target appeared was manipulated. Under the “L-rate” practice schedule, as in the standard epoch, the number of trials of the learned target was equal to that of each of the other targets. Therefore, it appeared just as often as the other targets (on average, once every 8 trials). However, under the “H-rate” practice schedule, the learned target appeared at a higher rate, four times more than each of the other targets. Whenever the learned target appeared, movement was executed under the force-field, which perturbed the hand perpendicular to its direction and proportional to its velocity.

The “L-rate learning set” was constructed as follows (Figure 1B, upper trace): the first to fourth days involved four successive epochs: (1) a default (standard) eight-target task (STD1, without force-field) of 80 trials; (2) a learning epoch (LRN, with force-field) of 240 trials (30 trials to the learned target); (3) a second default eight-target task (STD2) of 80 trials and (4) a second learning epoch (LRN2) of at least 240 trials. The fifth day involved only three consecutive epochs: (1) STD1; (2) LRN and (3) a long STD to negate the learning effect ("washout") of 360–480 trials. No cue was given to the monkey to mark the transitions between standard and learning epochs.

Note that the number of default trials in STD1 and STD2 were introduced with caution. The default trials can show the baseline condition on each day and the post-learning effects. However, they can interfere with the learning since they have a washout effect. We chose the number of trials in STD1 and STD2 to balance the trade-off between these two effects: we kept the number of default trials small enough to minimize the interference and large enough to measure the behavior and neural activity in the default condition. STD1 allowed us to examine the baseline for each day, before additional learning took place. For example, it made it possible to estimate the directional tuning of all cells without perturbation, as well as the overnight retention of learning. STD2 provided a rapid assessment of the learning effect without the perturbation present, with minimal washout. On days five, STD2 was replaced by the washout STD epoch to negate the learning effect.

The “H-rate learning set” consisted of only two days, since each day included four times as many trials of the learned target. As depicted in figure 1B (lower trace) the first day was identical to the first day of L-rate with a STD1-LRN-STD2-LRN2 structure, where the LRN epoch constituted 120 trials of the learned target and 30 of each of the seven non-learned targets. The second day was similar to the fifth day of the L-rate, with a washout as the third and last epoch.

Note that (i) each monkey experienced both L-rate and H-rate schedules at different sets. (ii) For each monkey, each set had a unique perturbation (learned target and FF direction) and (iii) learned targets that were experienced under L-rate were different than those experienced under H-rate.

Figure 1A illustrates adaptation to force-field in which the learned target was 0° and the force-field was clockwise. The force-field was applied only during the learning epoch and only to this target. Monkeys were trained for several months on the default eight-target task but were not exposed to the force-field prior to the recordings.

### B. Data analysis

Cells were recorded and sorted online by the Cyberkinetics online spike sorter. In cases of doubts (this was in about 10–15 electrodes, out of the 96 electrodes in the array, in each day of recording), we ran off-line spike sorting on the data and re-sorted the spikes. We selected single neurons for analyses that were recorded during one of these days and met five inclusion criteria: (i) well-isolated spikes; (ii) stable recordings based on firing rates in the first hold epoch throughout all trials; (iii) a significant effect for direction (one way ANOVA, p<0.01); (iv) a cosine fit (r(d) = a+b*cos(d-d_0_)) for directional tuning [*62*] that exceeded R^2^ = 0.65; and (v) a firing rate above 3 Hz.

The neuronal ensemble consisted of all the neurons recorded simultaneously during each session.

#### Behavioral performance during learning

The deviations in trajectories were assessed by the angular directional deviation of the hand from the target direction150 ms after movement onset. See learning curves in Figure 2.

#### Changes in firing rate of single cells

The firing rates were computed around movement onset, 200 ms prior to 300 ms post movement onset. For each cell we computed the following changes in activity:

1. For each perturbed trial of the learned target, we calculated the fraction of change in activity in this trial as compared to the average activity in unperturbed trials to the learned target during STD1.

2. For each unperturbed trial of the learned target during the washout epoch, we computed the fraction of change in activity in this trial as compared to the average activity in the last 10 force-field trials before washout.

Given x, the average change in activity for a given subgroup of cells along a given epoch of trials, we fit a two-exponential function: f(x) = a*exp(b*x) +c*exp(d*x), using the nonlinear least squares method.

#### Drop index

Given the average deviation of trajectories in the first 3 force-field trials that were executed immediately after standard epoch STD2 (e.g. first trials of LRN2) and the average deviation in the 5 trials just before STD2 (e.g. last trials of LRN), we computed the drop index as the difference between these average deviations, divided by their sum. Therefore, the drop index is positive if deviations were larger after STD2 epoch than before it.

Similarly, for each cell we computed the drop index between its firing rates in the trials before and after STD2.

#### Preferred direction (PD) analysis

Given the 8 average firing rates for the 8 movement directions during STD1 epoch, PDs were computed by a cosine fit (r(d) = a+b*cos(d−d_0_)) [*62*] , where the attributed angles were the average initial hand movement directions to each of the target directions.

Data includes both learning sets of CW and CCW curl fields. Therefore, in all of the analyses and figures, CCW was inverted to correspond to CW fields. Specifically, positive deviations were in the force-field direction, and negative deviations were counter to the force-field.

In all figures, error bars indicate standard error of the mean (SEM). Asterisks show 1% significance, using the Holm-Bonferroni method to correct for multiple comparisons.

## References

[1] Ghez C, Sainburg R (1995). Proprioceptive control of interjoint coordination.. Can J Physiol Pharmacol.

[2] Li CS, Padoa-Schioppa C, Bizzi E (2001). Neuronal correlates of motor performance and motor learning in the primary motor cortex of monkeys adapting to an external force field.. Neuron.

[3] Arce F, Novick I, Mandelblat-Cerf Y, Zvi I, Ghez C (2010). Combined Adaptiveness of Specific Motor-Cortical Ensembles Underlies Learning.. J Neurosci.

[4] Gandolfo F, Mussa-Ivaldi FA, Bizzi E (1996). Motor learning by field approximation.. Proc Natl Acad Sci U S A.

[5] Bays PM, Flanagan JR, Wolpert DM (2005). Interference between velocity-dependent and position-dependent force-fields indicates that tasks depending on different kinematic parameters compete for motor working memory.. Exp Brain Res.

[6] Izawa J, Rane T, Donchin O, Shadmehr R (2008). Motor adaptation as a process of reoptimization.. J Neurosci.

[7] Emken JL, Benitez R, Sideris A, Bobrow JE, Reinkensmeyer DJ (2007). Motor adaptation as a greedy optimization of error and effort.. J Neurophysiol.

[8] Lackner JR, DiZio P (1994). Rapid adaptation to Coriolis force perturbations of arm trajectory.. J Neurophysiol.

[9] Shadmehr R, Mussa-Ivaldi FA (1994). Adaptive representation of dynamics during learning of a motor task.. J Neurosci.

[10] Paz R, Boraud T, Natan C, Bergman H, Vaadia E (2003). Preparatory activity in motor cortex reflects learning of local visuomotor skills.. Nat Neurosci.

[11] Arce F, Novick I, Vaadia E (2005). Facilitation rather than interference in sequential adaptation to kinematic and dynamic perturbations.

[12] Pascual LA, Nguyet D, Cohen LG, Brasil NJ, Cammarota A (1995). Modulation of muscle responses evoked by transcranial magnetic stimulation during the acquisition of new fine motor skills.. J Neurophysiol.

[13] Nudo RJ, Milliken GW, Jenkins WM, Merzenich MM (1996). Use-dependent alterations of movement representations in primary motor cortex of adult squirrel monkeys.. J Neurosci.

[14] Karni A, Meyer G, Rey HC, Jezzard P, Adams MM (1998). The acquisition of skilled motor performance: fast and slow experience-driven changes in primary motor cortex.. Proc Natl Acad Sci U S A.

[15] Newell KM, Liu YT, Mayer-Kress G (2001). Time scales in motor learning and development.. Psychological Review.

[16] Newell KM, Mayer-Kress G, Lee Hong S, Liu Y (2009). Adaptation and learning; Characteristic timescales of performance dynamics.. Human Movement Science.

[17] Fusi S, Asaad WF, Miller EK, Wang XJ (2007). A neural circuit model of flexible sensorimotor mapping: learning and forgetting on multiple timescales.. Neuron.

[18] Tamashita Y, Tani J (2008). Emergence of functional hierarchy in a multiple timescale neural network model: A humanoid robot experiment.. PLoS Comput Biol.

[19] Smith MA, Ghazizadeh A, Shadmehr R (2006). Interacting adaptive processes with different timescales underlie short-term motor learning.. PLoS Biol.

[20] Sainburg RL, Ghez C, Kalakanis D (1999). Intersegmental dynamics are controlled by sequential anticipatory, error correction, and postural mechanisms.. J Neurophysiol.

[21] Donchin O, Francis JT, Shadmehr R (2003). Quantifying generalization from trial-by-trial behavior of adaptive systems that learn with basis functions: theory and experiments in human motor control.. J Neurosci.

[22] Thoroughman KA, Shadmehr R (2000). Learning of action through adaptive combination of motor primitives.. Nature.

[23] Mattar AAG, Ostry DJ (2007). Modifiability of Generalization in Dynamics Learning.. Journal of Neurophysiology.

[24] Paz R, Nathan C, Boraud T, Bergman H, Vaadia E (2005). Acquisition and generalization of visuomotor transformations by nonhuman primates.. Exp Brain Res.

[25] Goodbody SJ, Wolpert DM (1998). Temporal and amplitude generalization in motor learning.. J Neurophysiol.

[26] Krakauer JW, Pine ZM, Ghilardi MF, Ghez C (2000). Learning of visuomotor transformations for vectorial planning of reaching trajectories.. J Neurosci.

[27] Hwang EJ, Donchin O, Smith MA, Shadmehr R (2003). A Gain-Field Encoding of Limb Position and Velocity in the Internal Model of Arm Dynamics.. PLoS Biol.

[28] Malfait N, Shiller DM, Ostry DJ (2002). Transfer of Motor Learning across Arm Configurations.. J Neurosci.

[29] Criscimagna-Hemminger SE, Donchin O, Gazzaniga MS, Shadmehr R (2003). Learned Dynamics of Reaching Movements Generalize From Dominant to Nondominant Arm.. Journal of Neurophysiology.

[30] Gandolfo F, Mussa-Ivaldi FA, Bizzi E (1996). Motor learning by field approximation.. Proc Natl Acad Sci U S A.

[31] Thoroughman KA, Taylor JA (2005). Rapid Reshaping of Human Motor Generalization.. J Neurosci.

[32] Huang VS, Shadmehr R (2007). Evolution of Motor Memory During the Seconds After Observation of Motor Error.. Journal of Neurophysiology.

[33] Mandelblat-Cerf Y, Novick I, Paz R, Link Y, Freeman S (2011). The neuronal basis of long term sensorimotor learning.. J Neurosci.

[34] Salinas E, Abbott LF (1994). Vector reconstruction from firing rates.. J Comput Neurosci.

[35] Aboukhalil A, Shelhamer M, Clendaniel R (2004). Acquisition of context-specific adaptation is enhanced with rest intervals between changes in context state, suggesting a new form of motor consolidation.. Neuroscience Letters.

[36] Commins S, Cunningham L, Harvey D, Walsh D (2003). Massed but not spaced training impairs spatial memory.. Behavioural Brain Research.

[37] Savion-Lemieux T, Penhune VB (2005). The effects of practice and delay on motor skill learning and retention.. Exp Brain Res.

[38] Bock O, Thomas M, Grigorova V (2005). The effect of rest breaks on human sensorimotor adaptation.. Exp Brain Res.

[39] Francis J (2005). Influence of the inter-reach-interval on motor learning.. Exp Brain Res.

[40] Huang VS, Shadmehr R (2007). Evolution of Motor Memory During the Seconds After Observation of Motor Error.. Journal of Neurophysiology.

[41] Thoroughman KA, Shadmehr R (2000). Learning of action through adaptive combination of motor primitives.. Nature.

[42] Scheidt RA, Dingwell JB, Mussa-Ivaldi FA (2001). Learning to Move Amid Uncertainty.. Journal of Neurophysiology.

[43] Wulf G, Schmidt RA (1997). Variability of practice and implicit motor learning.. J Exp Psychol Learning Mem Cog.

[44] Cunningham HA, Welch RB (1994). Multiple concurrent visual-motor mappings: Implications for models of adaptation.. J Exp Psych: Hum Percep and Perf.

[45] Robertson EM, Pascual-Leone A, Miall RC (2004). Current concepts in procedural consolidation.. Nat Rev Neurosci.

[46] Shea CH, Kohl RM (1990). Specificity and Variability of Practice.. Res Q Exerc Sport.

[47] Simon DA, Bjork RA (2001). Metacognition in motor learning.. J Exp Psychol Learning Mem Cog.

[48] Imamizu H, Sugimoto N, Osu R, Tsutsui K, Sugiyama K (2007). Explicit contextual information selectively contributes to predictive switching of internal models.. Exp Brain Res.

[49] Monsell S (2003). Task switching.. Trends in Cognitive Sciences.

[50] Todorov E (2004). Optimality principles in sensorimotor control.. Nat Neurosci.

[51] Todorov E, Jordan MI (2002). Optimal feedback control as a theory of motor coordination.. Nat Neurosci.

[52] Brashers-Krug T, Shadmehr R, Bizzi E (1996). Consolidation in human motor memory.. Nature.

[53] Shadmehr R, Holcomb HH (1997). Neural correlates of motor memory consolidation.. Science.

[54] Shadmehr R, Brashers-Krug T (1997). Functional stages in the formation of human long-term motor memory.. J Neurosci.

[55] Donchin O, Sawaki L, Madupu G, Cohen LG, Shadmehr R (2002). Mechanisms influencing acquisition and recall of motor memories.. J Neurophysiol.

[56] Caithness G, Osu R, Bays P, Chase H, Klassen J (2004). Failure to consolidate the consolidation theory of learning for sensorimotor adaptation tasks.. J Neurosci.

[57] Krakauer JW, Ghez C, Ghilardi MF (2005). Adaptation to visuomotor transformations: consolidation, interference, and forgetting.. J Neurosci.

[58] Goedert KM, Willingham DB (2002). Patterns of Interference in Sequence Learning and Prism Adaptation Inconsistent With the Consolidation Hypothesis.. Learning & Memory.

[59] Brashers-Krug T, Shadmehr R, Bizzi E (1996). Consolidation in human motor memory.. Nature.

[60] Krakauer JW, Pine ZM, Ghilardi MF, Ghez C (2000). Learning of visuomotor transformations for vectorial planning of reaching trajectories.. J Neurosci.

[61] Poggio T, Bizzi E (2004). Generalization in vision and motor control.. Nature.

[62] Georgopoulos AP, Kalaska JF, Caminiti R, Massey JT (1982). On the relations between the direction of two-dimensional arm movements and cell discharge in primate motor cortex.. J Neurosci.

